# Mosquito Control Strategies and Insecticide Resistance of the Malaria Vector in Urbanized Land Use Types in Suame Municipality, Ghana

**DOI:** 10.1155/2024/5843481

**Published:** 2024-08-01

**Authors:** Jennifer Oppong, Silas Wintuma Avicor, Philip Kweku Baidoo, Patrick Addo-Fordjour, John Asiedu Larbi, Carlos Frimpong Akowuah, Akosua Boateng, Isaac Prince Essien, Gloria Mensah

**Affiliations:** ^1^ Department of Theoretical and Applied Biology Faculty of Biosciences College of Science Kwame Nkrumah University of Science and Technology, Kumasi, Ghana; ^2^ Entomology Division Cocoa Research Institute of Ghana, New Tafo-Akim, Ghana

## Abstract

Modified landscapes could create breeding habitats for mosquitoes and affect their community structure and susceptibility with implications for their management. Hence, in this study, household mosquito control methods in two urbanized landscapes; industrial and residential human settlements, in Ghana and insecticide susceptibility of the inhabiting *Anopheles* populations were assessed. Household knowledge and usage pattern of mosquito control methods in the modified landscapes were obtained using a questionnaire. Female adult *Anopheles* mosquitoes were also subjected to susceptibility tests using mosquito coils (0.08% meperfluthrin, 0.03% dimefluthrin, and 0.3% transfluthrin) and World Health Organization (WHO) insecticide test papers (0.05% deltamethrin, 4% DDT, 0.1% bendiocarb, 0.25% pirimiphos-methyl, and 5% malathion). Although insecticide-treated nets and aerosols were used for mosquito control, mosquito coils were the most common and widely preferred household method. The *Anopheles* mosquitoes were resistant to all the insecticides (mosquito coils and WHO test papers) except pirimiphos-methyl. Land use type did not affect insecticide resistance, but the insecticide type did. The findings indicate the effect of household insecticide usage practices on local mosquito populations and their implications for effective vector management and disease control in modified landscapes.

## 1. Introduction

Population increase leads to the creation of space for human settlement and industrialization, resulting in landscape modification. This alters the environment and may result in the establishment of habitats that promote the proliferation of mosquitoes [1, 2]. Hence, mosquitoes in contrasting land use types may respond differently to control measures including insecticides due to the adaptation to contaminants in their breeding habitats [3–7] which may be occasioned by the modified landscapes. This could impact on the choice of mosquito control method as well as the usage pattern, effectiveness [8] and eventually disease prevention and control.

Despite global improvements in vector control, mosquito-borne diseases still pose economic, health, and developmental challenges in many countries. Among these, malaria is one of the most fatal, causing about 608,000 deaths globally from an estimated 249 million cases in the year 2022 [9]. Malaria, which is vectored by *Anopheles* mosquitoes, is an endemic and recurrent disease in Ghana with the entire population at risk. Controlling the vectors is an important strategy in curbing the disease. To achieve this, various techniques including insecticide use, avoidance of mosquito-infested habitats, use of barriers like nets or screens, and environmental modification are employed [10, 11].

In Ghana, the routinely used control methods in households include insecticide-treated (mosquito) nets (ITNs), mosquito coils, creams, and aerosols [12, 13]. However, mosquito coils are among the most preferred, preventing the entry of mosquitoes into houses as they create barriers which hinder host-seeking activity of the vectors [14], impair their ability to land and feed on hosts [15], and kill them. The usage of mosquito coils is influenced by several factors including perceived effectiveness, acceptance, and cost [13]. Studies, however, indicate that these coils are ineffective in combating mosquitoes as a sole control strategy and may have some adverse effects on humans [8, 13, 16, 17].

Although several factors [13, 18] drive household usage of domestic insecticides, the frequency of use may be due to the need to avoid mosquito nuisance since the sole use of some of these products is ineffective in preventing mosquito bites [8]. The acceptance and efficacy of a mosquitocide may affect its usage pattern, and this could in turn impact on the environment, humans, and the target mosquitoes. The usage pattern may however depend on the land use type due to the inherent vector burden of the modified landscape. Mosquito diversity, behaviour, and response to insecticides could vary in different environments [4, 8, 19–21] such as the differential mortality levels observed in *Anopheles gambiae* populations from residential and industrial areas in Nigeria [22]. Pyrethroid resistance in *Anopheles* mosquitoes in some residential urban areas in Ghana was also related to the high insecticide residues in their breeding habitats [4]. Another study [8] in urban sites in southern Ghana also associated *Culex* insecticide resistance with household mosquito control strategies. It is therefore important to assess household practices regarding mosquito control and the efficacy of the most commonly used methods to inform vector management in different land use types. The exposure to household insecticides and the associated pesticide challenge in the modified landscapes may also contribute to resistance to nonhousehold insecticides such as active ingredients recommended by the World Health Organization (WHO) for indoor residual spraying (IRS). Hence, this study evaluated the household mosquito control methods in different land use types in Ghana and the insecticide susceptibility of the inhabiting *Anopheles* mosquitoes.

## 2. Materials and Methods

### 2.1. Study Area

The study was conducted in 2 different urbanized land use types in the Suame Municipality [21] of the Ashanti region of Ghana. Suame Magazine (06°43′30″ N 1°38′30″ W) is an auto-mechanic industrialized area with a residential and a nonresidential population of about 49,095 and 200,000, respectively, while Bremang (06°43′60″ N 1°38′60″ W) is a residential area with a population of 91,005 [23]. The study area is situated in the semideciduous forest zone and has two rainy periods (March to July and September to November) with a temperature range of 21.5 to 30.7°C and relative humidity of 83.2%.

### 2.2. Ethics Approval

Ethical clearance was obtained from the Committee on Human Research, Publication and Ethics (Ref: CHRPE/AP/334/19) of the Kwame Nkrumah University of Science and Technology and the Suame Municipal Assembly (Ref: SMA/GA.1/14/01/10).

### 2.3. Knowledge, Attitude, and Perception of Insecticide Usage of Inhabitants

A total of 171 and 326 questionnaires were administered in the industrial and residential area, respectively. The industrial area was demarcated into 23 zones and 17 were selected for the sampling because the other zones had no residents. Houses within the 17 zones were numbered and the questionnaires (171) were distributed among the zones. In each zone, questionnaires were randomly administered based on the numbered houses. A similar process was conducted in the residential area. However, in Bremang, the 326 questionnaires were distributed among 3 suburbs within the locality (thus Bremang Fie, Bremang Universal Gospel Center, and Bremang Nkontwima) which are well laid out with numbered houses. The questionnaire was used to obtain information on the demography, socioeconomics, knowledge, perception, control methods, and usage pattern of mosquito control methods of the households.

### 2.4. Mosquito Sampling and Processing

Mosquito larvae were sampled from diverse breeding habitats (excavator bucket, streams, gutters, tyre casing, and metal containers) in the study area [21] from June to October 2019. These were sorted into their respective genera [21] and *Anopheles* larvae were transferred into disposable cups (6.2 × 6.0 cm) containing 100 ml of water. The cups were placed in 40 cm^3^ cages (1 cup per cage) and the larvae were fed with Raanan® fish pellet daily before pupation. Afterwards, female *Anopheles* that emerged was used for the bioassay tests. The rearing temperature and humidity were between 25 and 30°C and 75–90%, respectively.

### 2.5. Mosquito Coil Bioassay

Efficacy of the commonly used mosquito coils (MC2: 0.03% dimefluthrin; MC6: 0.3% transfluthrin; and MC7: 0.08% meperfluthrin) in the households based on the questionnaire data was assessed in an exposure chamber (4 × 4 × 2.12 m; length × breadth × height). Twenty sugar-fed (10% sucrose solution on cotton wool) female [24] *Anopheles* mosquitoes (2–5 days old) were aspirated into 4 cages (30 × 30 × 30 cm). Each cage was hanged in the corner of the chamber at 20 cm from the ceiling and approximately 10 cm from the wall. The mosquitoes were held for an hour before the coil was lit. A fan (Nasco™, China, diameter of head = 45.7 cm) at its minimum wind speed was placed in the middle of the chamber to circulate smoke emitted by the coil. Facing upwards, the fan was placed on the floor and a plate with the coil was placed beside it. The mosquito coil was lit to allow smoke emission before the fan was switched on. The doors and windows of the exposure chamber were closed during the experiment. The number of mosquitoes that were knocked down within 60 min was recorded. Afterwards, the mosquitoes were transferred from the cages into the holding tubes and fed *ad libitum* with cotton wool soaked in 10% sugar solution. The mosquitoes were held at 27 ± 2°C and 80 ± 10% relative humidity for 24 h and the observed mortality was recorded. The test was replicated three times per mosquito coil. The control experiment was conducted per this procedure; however, no coil was used [24].

### 2.6. WHO Insecticide Susceptibility Test

Insecticide susceptibility of the female mosquitoes was determined using the WHO [25] standard tube assay. Five insecticide-impregnated papers from four insecticide classes approved by the WHO for IRS were used for the test. These were 0.05% deltamethrin (pyrethroid), 4% DDT (organochlorine), 0.1% bendiocarb (carbamate), 0.25% pirimiphos-methyl (organophosphate), and 5% malathion (organophosphate). Nonblood (10% sugar solution soaked on cotton wool) fed female *Anopheles* mosquitoes aged 2–5 days were exposed to each insecticide-impregnated paper and a control. Mosquitoes were exposed to the insecticides for 60 min and within that period, knockdown was recorded at 5 min intervals for 20 min and then at 10 min intervals for the rest of the 40 minutes. Mortality was recorded after 24 hours. The test was replicated 4 times with 20 mosquitoes each.

### 2.7. Data Analysis

Questionnaire data was inputted into Excel, exported to SPSS ver. 23 and analyzed. The % knockdown (%KD) was computed as the percentage of the number of knocked down mosquitoes compared to the total number of exposed mosquitoes per insecticide. Two-way analysis of variance (ANOVA) was used to compare the effect of land use type and mosquito coil brand on mosquito mortality. The interaction effect of the two factors on mosquito mortality was also tested. Tukey HSD post hoc test was used to separate the means. ANOVA was run at a significance level of 5%.

## 3. Results

### 3.1. Knowledge and Usage of Household Mosquito Control Strategies

Many of the respondents in both land use types were females (Table 1). Majority (82.4%: industrial; 83.1% residential) of the respondents had basic and senior high school education and many of the respondents (91.8%: industrial; 96%: residential) were employed.

Mosquito coil and repellent cream had the highest and lowest usage, respectively, in the study area (Table 2). Other mosquito control items used by respondents included mosquito spray/aerosol and insecticide-treated net (ITN) while a small number of the respondents (1.8%: industrial; 1.2%: residential) did not use any control method. Some unconventional methods such as burning herbs and the use of an electric fan were also employed (Table 2). In the industrial area, all respondents who use multiple mosquito control methods combined mosquito coil with other methods while 53.8% (43/80) of multiple control method users in the residential area use mosquito coil in combination with other methods (Tables 2 and 3).

Users of mosquito coils preferred specific brands (Table 3). The brands were coded in this study using the prefix MC (mosquito coil) and a number to avoid disclosing their identities. A few respondents (2.1%) in the industrial area used any brand/type that was available. Many respondents at the residential area preferred MC7 to the other brands.

### 3.2. Pattern of Mosquito Coil Usage and Perception of Side Effects

Majority (65.5% industrial; 70.9% residential) of the coil users used at least one coil daily (Table 4). The coils were usually used only indoors or both indoors and outdoors with many (80% industrial; 70.3% residential) of the respondents sleeping in a surrounding with a lit coil. Many (64.8% industrial; 51.7% residential) respondents reported that coils repel mosquitoes rather than kill them. At the industrial area, 65.5% reported that mosquito coils had some effects on humans while 34.6% of the respondents were not sure of the side effects of coils on humans (Table 5). In the residential area, about 79% of the respondents stated that coils had adverse effects on humans (Table 5) with some of the effects being cold, catarrh, cough, skin/eye irritation and breathing difficulty. While cold and cough were the joint highest reported (17.9% each) in the industrial area, catarrh was the highest reported (33.7%) in the residential area. Skin/eye irritation was the least reported (2.1%) in the industrial area while choking (0%) was not reported in the residential area.

### 3.3. Perception of Mosquito Coil Effectiveness at the Auto-Mechanic Industrial and Residential Areas

Many respondents (77.2%: industrial; 73.8%: residential) perceived mosquito coils to be effective and used them frequently (68.3%: industrial; 60.9%: residential) due to their effectiveness compared to their affordability, which was about 2-fold lower (Table 6).

### 3.4. Mosquito Coil Efficacy

In both land use types, *Anopheles* knockdown after 60 min was highest with MC6 and lowest with MC2 (Table 7). None of the mosquitoes in the control was knocked down after 60 min. After 24 h, mosquito mortality in the control was less than 5% (2.50 ± 1.25%: industrial; 3.33 ± 1.10%: residential). There was a 30–67% mosquito mortality when exposed to the mosquito coils (Table 7). There was no significant influence of land use type on *Anopheles* mortality (*F*_1,16_ = 0.279, *p*=0.603), but the type of mosquito coil significantly affected *Anopheles* mortality (*F*_3,16_ = 581, *p*=0.001). The interaction effect of land use type and mosquito coil on mosquito mortality was significant (*F*_3,16_ = 2.85, *p*=0.007). The performance of each mosquito coil was similar within the two land use types, except MC6 which recorded a higher and significantly different mortality at the residential area compared to the auto-mechanic area (*p* < 0.05).

### 3.5. WHO Insecticide Susceptibility

**Figure 1:**
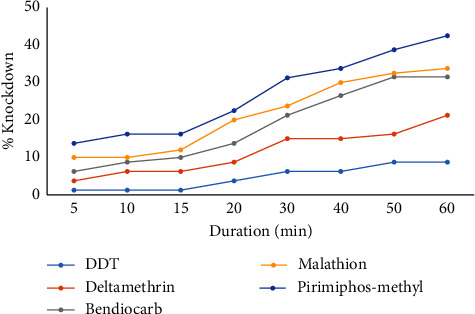
Knockdown of *Anopheles* from the industrial area after insecticide exposure. The insecticide concentrations were 0.05% (deltamethrin), 4% (DDT), 0.1% (bendiocarb), 0.25% (pirimiphos-methyl), and 5% (malathion).

**Figure 2:**
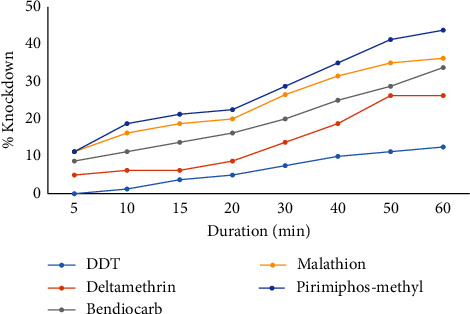
Knockdown of *Anopheles* from the residential area after insecticide exposure. The insecticide concentrations were 0.05% (deltamethrin), 4% (DDT), 0.1% (bendiocarb), 0.25% (pirimiphos-methyl), and 5% (malathion).

Among the 5 insecticide papers, pirimiphos-methyl had the highest % knockdown after 5 min of exposure (Figures 1 and 2) and this trend continued to the 60^th^ min (42.5%: industrial; 43.75%: residential). DDT recorded the lowest % knockdown (8.75%: industrial; 12.5%: residential) on the mosquitoes. For all the insecticides, the % knockdown increased rapidly from the 15^th^ to the 60^th^ min.

*Anopheles* mosquitoes in both communities were resistant to the insecticides tested (<90% mortality) except pirimiphos-methyl (≥98% mortality). Mortality of *Anopheles* mosquitoes after exposure to the insecticides did not vary significantly between the industrial and residential areas (*F*_1,36_ = 0.85, *p*=0.362) (Table 8). However, mortality of *Anopheles* mosquitoes differed significantly among the various insecticides used (*F*_5,36_ = 161.06, *p*=0.001). There was no interaction effect of land use type and insecticide on *Anopheles* mortality (*F*_5,36_ = 0.048, *p*=0.996). Significant differences occurred among all the insecticide type pairs except for deltamethrin–bendiocarb, and malathion–bendiocarb pairs (Table 8). Pirimiphos-methyl killed significantly more mosquitoes than any of the insecticides while DDT was the least effective insecticide.

## 4. Discussion

Although households in the study area use different mosquito control methods, mosquito coil usage was the most prevalent in both land use types, an observation reported in communities in the Ashanti, Bono East, Central, Greater Accra, Upper East, and Western regions in Ghana [8, 12, 13, 17]. This can be attributed to factors such as its perceived effectiveness, availability, and affordability [13] compared to the other control methods. The median cost of spatial repellent to protect one person per year is similar to that of ITN and lower than that of IRS [11]. Usage of a combination of mosquito control methods was high, and this usually revolved around the use of mosquito coils with the other methods. This demonstrates the integral role of mosquito coils in household mosquito control and the inability of a single household method to confer the desired antimosquito protection, hence augmenting with other methods to increase the level of protection [13, 16, 26].

Mosquito coil usage was mostly on a daily basis in both land use types. This is similar to findings in other parts of Ghana [12, 13, 17]. The daily usage is suggestive of the persistent threat posed by mosquitoes in the study area and the constant need for protection. Although most coil users usually lit one coil, the proportion of people who used more than one coil was higher in the industrial area. This probably indicates a higher mosquito threat in the industrial area since our previous study [21] observed a higher mosquito abundance in this land use type. While most of the residential households used coils indoors, those in the industrial area used them in both indoor and outdoor environments. It is likely that the mosquitoes in the residential area are largely endophagous and endophilous while in the industrial area, there is a sympatry of both indoor and outdoor feeding and resting mosquitoes.

Most coil users sleep in the presence of a burning coil. This practice is common in several countries [26–28] and exposes users to coil emissions. Although Hogarh et al. [26] estimated a low environmental health risk associated with the usage of some coils, coil users in our study reported adverse effects on their skin, eyes, and respiratory function. Such effects have also been reported in other studies [12, 13, 17, 28, 29]. Mosquito coils are advertised as inducers of mosquito death; however, most users regarded them as effective in repelling rather than killing mosquitoes [12, 13, 17]. Users generally regarded the coils as effective in mosquito avoidance and the effectiveness based on this attribute was the most predominant reason for its usage.

Among the mosquito coils used, 0.3% transfluthrin induced a higher knockdown and mortality than the others. This is similar to a study by Bibbs et al. [30] where transfluthrin and meperfluthrin were observed to have the highest vapour toxicity on some mosquito species compared to other volatile pyrethroids. Although majority of the respondents cited effectiveness of coils in controlling mosquitoes as the reason for their usage, our results show that the *Anopheles* mosquitoes were resistant to the insecticide coils. This result is consistent with previous reports of *Anopheles* resistance to mosquito coils [12, 13, 16, 26]. Hence, this relates to the users' perception of repellency and not mortality as the most predominating coil property.

Although the land use type did not influence mosquito mortality, the coil type did, with 0.3% transfluthrin inducing the highest mortality. Mosquito coil brands used in the industrial area were more than the residential area. This could be due to the experimentation of different coil types by households in the quest for the most effective in the industrial area. Differential mortality effect on the mosquitoes may be due to the type of active ingredient and resistance to the insecticide coils could be attributed to the excessive and indiscriminate use of domestic insecticides in the study area. Exposure to heavy metal pollutants can increase insecticide resistance in mosquitoes [5]; hence, metal pollutants and other contaminants in water of mosquito breeding sites within the industrial area could have partly contributed to the lower mortality.

The WHO insecticide test results indicated that for each insecticide, mosquito mortality from the residential area was higher than the industrial area although this was not significant between the two sites. A similar pattern in mosquito mortality was observed between *Anopheles gambiae* populations from industrial and residential areas in Kano, Nigeria for malathion and bendiocarb [22]. Also, except pirimiphos-methyl, mosquitoes from the two land use types in our study were resistant to the insecticides. Mortality of the mosquitoes to the insecticides was significantly different in each land use type, although the mortality response to bendiocarb was not significantly different from those to deltamethrin and malathion. Resistance of *Anopheles* mosquitoes to insecticides has been reported around the country over the past decades [31–34], and these insecticides continue to be ineffective in controlling mosquitoes as observed in our study. Most of these previous studies were conducted in agricultural areas, thereby attributing mosquito resistance to chemical contamination during agricultural activities. Hunt et al. [32] detected *Anopheles* resistance/suspected resistance to 4 classes of insecticides (pyrethroids, carbamates, organophosphates, and organochlorines) in four mining areas in Ghana although susceptibility was detected to one organophosphate active ingredient. Similarly, our study shows resistance of *Anopheles* mosquitoes to active ingredients in the four insecticide classes (i.e., <90% mortality) except the organophosphate pirimiphos-methyl. Resistance to bendiocarb and DDT have also been observed in modified landscapes such as industrial and residential sites in Nigeria [22] and different agricultural production sites in Cote D'Ivoire [35]. However, the *Anopheles* mosquitoes in the study in Nigeria were susceptible to malathion [22] unlike the populations in our study and that of two vegetable cultivation sites in Kouadio et al. [35].

The questionnaire responses showed frequent use of ITNs, aerosols, and mosquito coils in the study area. This practice might have contributed to pyrethroid resistance [4, 8] due to the selection pressure. Continuous exposure of mosquitoes to pyrethroid insecticides can induce metabolic enzymes [36, 37], potentially resulting in the development of resistance. Insecticide contamination of mosquito breeding habitat has also been implicated in mosquito resistance in an urban residential area with no agricultural activity [4]. Although, the current study did not focus on associating resistance with breeding site contamination, it is important to explore this phenomenon to gain more understanding in this regard. The susceptibility of *Anopheles* mosquitoes to the organophosphate insecticide (pirimiphos-methyl) in our study substantiates previous findings [32–34]. Thus, given the resistance of the mosquitoes to pyrethroids, the organophosphate pirimiphos-methyl can be used as an alternative in Indoor Residual Spraying.

## 5. Conclusion

Mosquito coil forms an important component of household antimosquito measures with diverse brands used at the auto-mechanic industrial area compared to the residential area. Even though coils were regarded as effective, mosquito repellency was the most widely reported effect compared to mosquito mortality. *Anopheles* mortality was not significantly affected by land use type despite the lower mortality at the industrial area. However, mortality was significantly affected by insecticide type. The mosquitoes were resistant to all the insecticides (including the mosquito coils) except pirimiphos-methyl. The current study contributes valuable information on *Anopheles* resistance towards insecticides for vector management, highlighting the potential impact of household insecticide use on resistance development and the implications for community-wide vector control using indoor residual spraying.

## Figures and Tables

**Table 1:** Demographic characteristics of respondents at the study area.

Category	Characteristics	Industrial area		Residential area	
		Frequency (*N* = 171)	(%)	Frequency (*N* = 326)	(%)
Gender	Male	73	42.7	150	46
	Female	98	57.3	176	54

Educational background	Basic	78	45.6	116	35.6
	Senior High School	63	36.8	155	47.5
	Tertiary	7	4.1	38	11.7
	No formal education	23	13.5	17	5.2

Occupation	Employed	157	91.8	313	96
	Not employed	14	8.2	13	4

*N*: number of respondents.

**Table 2:** Household mosquito control methods at the study area.

Control method	Industrial area		Residential area	
	Frequency (*N* = 171)	(%)	Frequency (*N* = 326)	(%)
ITN	7	4.1	68	20.9
Mosquito coil (only)	77	45	129	39.6
Mosquito spray	10	5.8	39	12
Multiple methods (MM)	68	39.8	80	24.5
Mosquito coil in MM	68	100	43	53.8
Repellent cream	0	0	3	0.9
No method	3	1.8	4	1.2
Others (fan and burning of herbs)	6	3.5	3	0.9

*N*: number of respondents.

**Table 3:** Mosquito coil brands used at the industrial and residential areas.

Brand	Active ingredient	Industrial area		Residential area	
		Frequency (*N* = 145)	(%)	Frequency (*N* = 172)	(%)
Any type	—	3	2.1	—	—
MC1	0.3% rich-d-trans allethrin	1	0.7	—	—
MC2	0.03% dimefluthrin	25	17.2	18	10.5
MC3	0.30% d-allethrin	1	0.7	—	—
MC4	0.33% d-allethrin	1	0.7	—	—
MC5	0.32% d-allethrin	—	—	10	5.8
MC6	0.3% transfluthrin	21	14.5	20	11.6
MC7	0.08% meperfluthrin	93	64.1	124	72.1

*N*: number of respondents.

**Table 4:** Frequency and pattern of mosquito coil usage at the auto-mechanic industrial and residential areas.

Category	Characteristics	Industrial area		Residential area	
		Frequency	(%)	Frequency	(%)
Coil usage	Daily	95	65.5	122	70.9
	Weekly	8	5.5	8	4.7
	Twice a week	32	22.1	19	11
	More than twice a week	9	6.2	15	8.7
	Occasionally	1	0.7	8	4.7

Number of coils lighted	1	68	46.9	158	91.9
	2	43	29.7	10	5.8
	3	26	17.9	3	1.7
	4	8	5.5	1	0.6
	>4	0	0	0	0

Place of coil usage	Indoors	68	46.9	142	82.6
	Outdoors	7	4.8	2	1.2
	Both	70	48.3	28	16.3

Sleep with coil	Yes	116	80	121	70.3
	No	19	13.1	48	27.9
	Sometimes	10	6.9	3	1.7

**Table 5:** Side effect perception of mosquito coil usage at the auto-mechanic industrial and residential areas.

Category	Characteristics	Industrial area		Residential area	
		Frequency	(%)	Frequency	(%)
Effect on mosquito	Repel	94	64.8	89	51.7
	Kill	41	28.3	70	40.7
	No effect	10	6.9	13	7.6

Effect on human	Cold	26	17.9	39	22.7
	Catarrh	24	16.6	58	33.7
	Cough	26	17.9	33	19.2
	Choking	9	6.2	0	0
	Skin/eye irritation	3	2.1	1	0.6
	All the above	7	4.8	5	2.9
	Not sure	50	34.6	36	20.9

**Table 6:** Effectiveness of mosquito coils and factors that influence usage.

Category	Characteristics	Industrial area		Residential area	
		Frequency	(%)	Frequency	(%)
Rating of coils	Effective	112	77.2	127	73.8
	Moderately effective	23	15.9	36	20.9
	Not effective	7	4.8	5	2.9
	Not sure	3	2.1	4	2.3

^*∗*^Reason for the frequent usage of coil	Effectiveness	99	68.3	103	60.9
	Affordability	46	31.7	66	39.1

^*∗*^No response from 3 respondents from the residential area.

**Table 7:** Susceptibility of *Anopheles* mosquitoes to pyrethroid-based mosquito coils.

Mosquito coil	Active ingredient	Industrial area		Residential area	
		% KD after 1 h	% mortality ± SE	% KD after 1 h	% mortality ± SE
MC2	0.03% dimefluthrin	0.8	30.00 ± 2.17^bA^	2.8	35.83 ± 1.10^cA^
MC6	0.3% transfluthrin	10	55.83 ± 2.20^aB^	11.3	67.08 ± 1.82^aC^
MC7	0.08% meperfluthrin	3.3	51.25 ± 1.44^aB^	4.6	53.75 ± 1.44^bB^
Control	—	0	2.50 ± 1.25^cD^	0	3.33 ± 1.10^cD^

KD: knockdown; SE: standard error. Values with different lowercase superscripts in the same column are significantly different (*p* < 0.05). Values with different uppercase superscripts are significantly different (*p* < 0.05) across columns and rows. Post hoc test was done using Tukey HSD.

**Table 8:** Mortality of mosquitoes 24 h after 1 h exposure to insecticides.

Insecticide	Industrial area		Residential area	
	Mortality ± SE (%)	^*∗*^RS	Mortality ± SE (%)	^*∗*^RS
Deltamethrin (0.05%)	55.00 ± 3.54^a^	R	58.75 ± 3.15^a^	R
Malathion (5%)	70.00 ± 6.45^b^	R	73.75 ± 4.27^b^	R
Bendiocarb (0.1%)	62.50 ± 4.33^ab^	R	63.75 ± 6.25^ab^	R
DDT (4%)	32.50 ± 1.44^c^	R	33.75 ± 6.25^c^	R
Pirimiphos-methyl (0.25%)	98.00 ± 1.44^d^	S	100.00 ± 0.00^d^	S
Control	2.00 ± 1.16^e^	—	3.00 ± 1.92^e^	—

SE: standard error. Values with different superscripts in the same column are significantly different (*p* < 0.05) as determined by Tukey HSD post hoc test. RS: resistance status. R: resistant. S: susceptible. ^*∗*^Based on the WHO susceptibility criteria [25].

## Data Availability

Data supporting the findings of this study are available upon reasonable request.
